# Declining Orangutans Population in the Unprotected Forest of Batang Toru

**DOI:** 10.21315/tlsr2018.29.2.6

**Published:** 2018-07-06

**Authors:** Arfah Nasution, Dyah Perwitasari-Farajallah, Sri Suci Utami-Atmoko

**Affiliations:** 1 Faculty of Mathematics and Natural Sciences, Bogor Agricultural University (IPB), Bogor 16680, West Java, Indonesia; 2 Primate Research Center, Bogor Agricultural University, Bogor, West Java, Indonesia; 3 Faculty of Biology, Universitas Nasional, Jakarta 12520, Indonesia

**Keywords:** Batang Toru, Declining Orangutan, East Batang Toru, Unprotected Forest, *Pongo tapanuliensis*

## Abstract

Habitat loss and hunting are major threats to the long-term survival of the viable orangutan population in Batang Toru. East Batang Toru Forest Block (EBTFB) is the most threatened area due to low forest cover and high encroachment. Based on a preliminary survey in 2008, Hopong forest which is located in EBTFB, had the highest orangutan density (0.7 ind/km^2^). However illegal logging and hunting of protected species were occuring in this unprotected forest. Since this location has been gazetted as unprotected forest from the first survey until this study was conducted, it is important to assess orangutans population trends. This study aims to provide an updated density of orangutan in Hopong forest. The study included the location of the original survey but covered a wider overall area. The line transect method was used to record orangutan nests, ficus and trees bearing fruits. A quadrat method was used to record vegetation. The encounter rate of orangutan declined from 0.7 ind/km^2^ to 0.4 ind/km^2^ between 2008 and 2015. Forest cover has also changed in the seven years between surveys and this has influenced orangutan and orangutan nest encounter rates in Hopong. Since unprotected forest is at more risk in comparison with protected forest, allocation status of the Hopong forest is critical to reduce the threats it faces.

## INTRODUCTION

The Tapanuli orangutan (*Pongo tapanuliensis*) is the third species of orangutan that discovered recently. This newly great ape is isolated in small numbers of fragments forest in Tapanuli – a district in North Sumatera (Nater *et al.* 2017) and the last population remaining to the south of Lake Toba (Wich *et al*. 2008). Since 2011, Nater *et al.* (2011) found that compared to the north of Lake Toba population, mtDNA of the Batang Toru population is more similar to Bornean orangutan.

Batang Toru forest is the last habitat units for this “rediscovered” orangutans (Meijaard 1997) in southernmost of Sumatra. The forest is divided into a western and an eastern forest block and administratively covers three districts of North Sumatra (North, Central and South Tapanuli). The Tapanuli orangutan is estimated to consist of 400 individuals in the west and 150 individuals in the east/East Sarulla (Wich *et al.* 2008). This estimation is based on a preliminary survey in the western forest block, while a guesstimate was made for the eastern forest block (Singleton *et al.* 2004). The first systematic survey of the orangutan population in East Batang Toru Forest Block (EBTFB) was carried out by Sumatran Orangutan Conservation Program (SOCP) – Batang Toru Program in 2008 (Fredriksson 2008).

Habitat availability in East Batang Toru is inadequate to harbour a large population of orangutan due to habitat loss and land conversion for settlement, geothermal and agriculture have caused the observed habitat loss (Onrizal & Perbatakusuma 2011). Another threat for long term survival of orangutan in Batang Toru is hunting (Wich *et al*. 2011). Hunting pressure has tended to be common in north-west of Batang Toru – including in East Sarulla, where human population is predominantly non-muslim (Wich *et al*. 2014). Combination of human population and hunting history have negative impact for orangutan population (Wich *et al*. 2016).

Higher number of orangutans in East Sarulla are found in Hopong. The forest in this area is relatively good with high level of food availability (Susanto *et al*. 2008). Unfortunately, this area is gazetted as unprotected forest which the deforestation rate is higher than in protected forest (Gregory *et al.* 2012). We assumed that orangutan population trend in unprotected forest tend to be decreasing due to high potential encroachment. This study aimed at determining an updated population density of orangutan in Hopong forest. We compare the condition of Hopong forest during the first survey (2008) with the present survey (2015) to find out the population trends in this area. These data are important as the guideline and basic data to improve the protected area of Batang Toru and establish protective management measures.

## METHODS

### Study Site

This study was conducted in Hopong Forest, East Sarulla, North Tapanuli, North Sumatra, Indonesia on 8–22 October 2015. The remaining forest in East Sarulla covers around 54,000 ha. The study site is in a forest around Hopong in the north of Dolok Sipirok Nature Reserve. The forest consisted of primary and secondary forest with a dense vegetation cover of Dipterocarpaceae, Sapotaceae, Fagaceae, Anacardiaceae, Lauraceae and Myrtaceae.

### Data Collection

The population density was measured by nest count using the line transect method following van Schaik *et al*. (1995). The transect design used systematic random sampling based on landsat image using Distance 5.0 and ArcGIS 9.3 (California, United States). Twelve transects were selected, each being 500 m in length and 500 m apart from each other which laid paralelly. The number and characteristics of detected nests was recorded. Recorded characteristics included: nest stage, nest position, nest height, nesting tree height, nesting tree diameter, nesting tree species, nest position from line transects and perpendicular distance (PPD). Nest stage was categorised into a four-class system: (a) fresh, some leaves still green; (b) nest is brown but remains intact; (c) leaves missing and holes appearing in nest; (d) leaves are gone, only branch structure of nest remains (van Schaik *et al.* 1995). Nest position was distinguished in five basic patterns which differ with respect to how the main platform is created (Prasetyo *et al*. 2009).

The distribution of orangutans was identified by recording the waypoint of the nest and overlaying these on a map of the study area. Threat level was estimated by measuring the distance between an encountered nest and the nearest road, settlement and agricultural area. Food availability as well as vegetation data were recorded during the survey. Food availability was recorded by measuring fruiting tree abundance and ficus density using fruit trail method (van Schaik *et al.* 1995). The ficus trees and the host trees of fallen fruits along the transects were recorded. The ficus were classified into two classes: Class I (ficus tree with living host), Class II (ficus tree with death host). Vegetation data was recoerded based on direct observation in the field using a sampling quadrat method. A total of 25 plots of 10 m × 10 m and 20 m × 20 m were chosen randomly. The tree’s diameter breast height in each plot was measured.

## DATA ANALYSIS

Following van Schaik *et al*. (1995), the basic equation for calculating nest density (D) from line transect surveys is: D = N/2wL, with nest density translated into orangutan density (d) using addition parameters: d = D/p × r × t in which: D = nest density (nest/km^2^), N = number of nest observed along transect, d = orangutan density (ind/km^2^), L = length of transect covered (km), p = proportion of nest builders in the population (0.9/day, van Schaik *et al.* 1995), r = rate at which nests are produced (1.22/day, SOCP 2016), t = time during which a nest remains visible (501.5 days, Wich *et al*. 2016), w = estimated width of the strip of habitat actually censused (km). w value is obtained from perpendicular distance which analysed using the computer package Distance^TM^ 5.0 (Buckland *et al*. 1993). Besides the nests within transects, nest encountered outside of transects were also recorded as additional data.

The spatial distribution map of orangutan nests was created using GPS waypoints which were overlayed on a basic map using the computer package ArcGIS 9.3. Fruit abundance was calculated with the formula: d = N/L, while ficus density was calculated with the formula: d = N/2wL. Habitat quality was analysed quantitatively for density, relative density, frequency, relative frequency, dominancy, relative dominancy, species importance value (SIV), Shannon-Wiener Diversity Index, and an orangutan food list.

## RESULTS

### Orangutan Distribution and Estimated Population Size

A total of 78 nests were recorded, of which 37 nests were found along a transect. This result was different from the first survey which detected orangutan directly and indirectly with 43 nests along a transects and 35 nests out of a transect (Susanto *et al*. 2008). Even though survey effort was improved by increasing the transects length (first survey: 4.025 km, updated survey: 6 km), number of nest encounters was less than in the first survey. This study showed a downward trend of orangutan population in Hopong. Orangutan population was declining from 0.7 ind/km^2^ (Susanto *et al*. 2008) to 0.4 ind/km^2^ in the past seven years. High numbers of nests were encountered beyond the transects at the edge of a ravine. A total of 42 nesting trees were detected, some of which consisted of two and three old nests. Fruit and ficus densities relatively low during this study; the previous study did not record these densities (Table 1).

**Figure 1:**
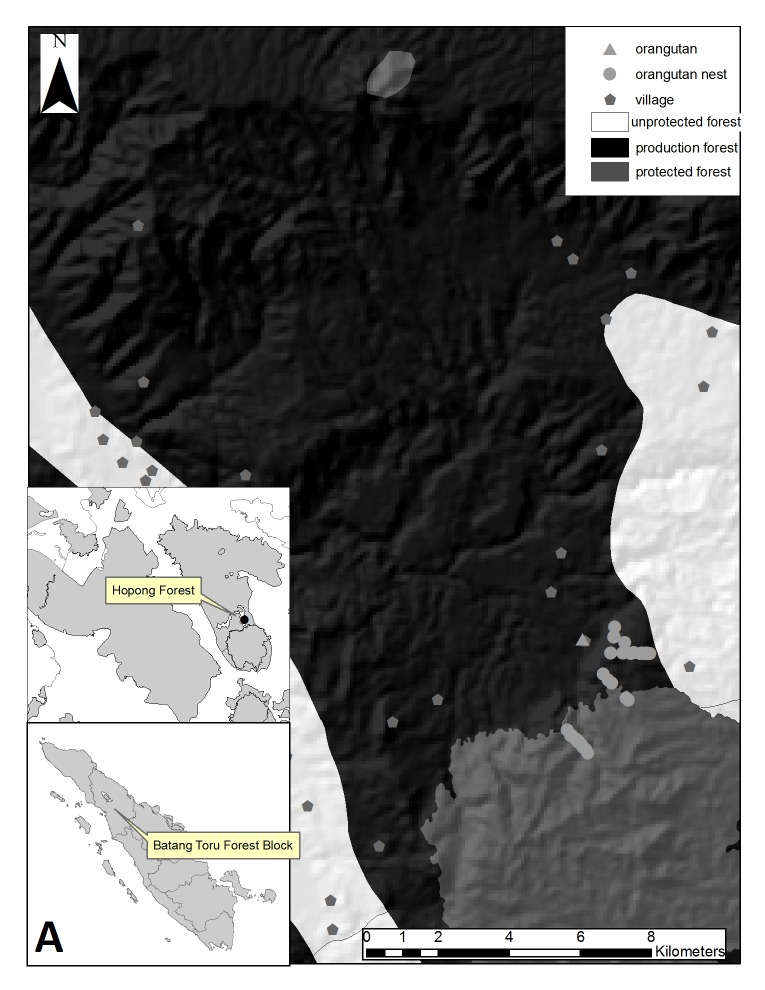

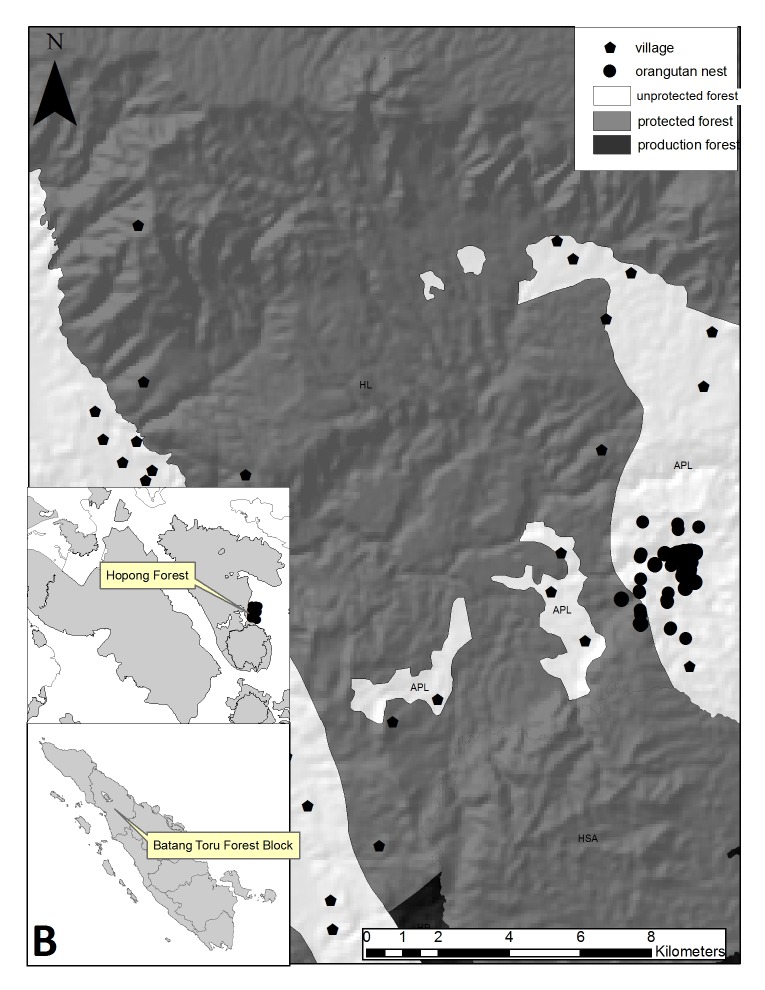
Map of orangutan nests in Hopong forest, East Sarulla in: (A) 2008 and (B) 2015.

Nests were predominantly located in the unprotected primary forest area. 85.7% of nests were found in the unprotected forest due to most of transects were predominantly laid in unprotected forest (only one transect laid in protected forest). Virtually, the entire of East Sarulla was gazetted as production forest in 2008. This area was gazetted into protected forest in 2014, but there was no change in the gazettment of Hopong forest. From 2008 to 2015 this area was still unprotected forest (Fig. 1).

### Threats

The nest distance to settlement, main road, and local agriculture was also changed in past seven years. The nest was found further from settlement, main road, and local agriculture, compared to nest distance in 2008. Result of Susanto *et al.* (2008), the nearest nest found from settlement and main road was at 0.1 km and 0 km respectively. On the other hand, the nearest nest found from settlement, main road, and local agriculture in the survey was 1.51 km, 1.14 km and 1.45 km.

### Vegetation Composition

**Figure 2:**
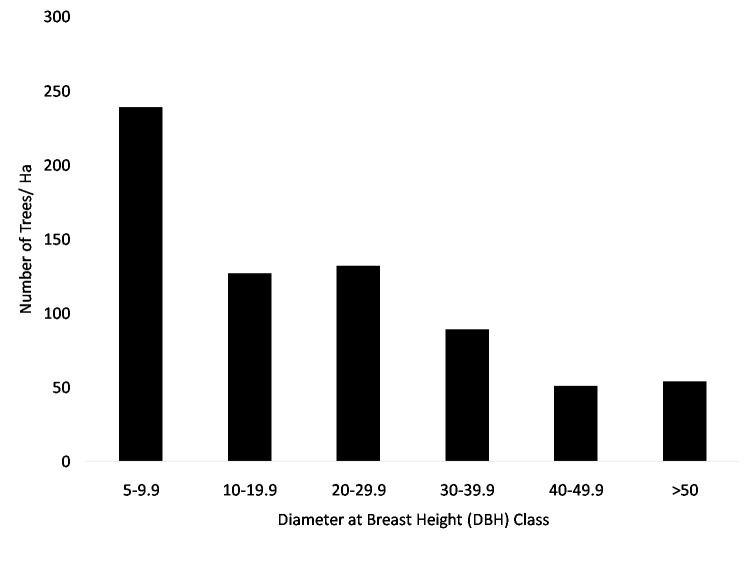
The distribution of class diameters in Hopong forests.

In total, 178 tree species were recorded in this study. Of these, 90 species were potential feeding trees for orangutan, and 39 species were potential nesting tree species. The species important value (SIV) were relatively low for each species (Table 2). The Shannon-Wiener diversity index of Hopong forest area is 4.6 for tree and 4.191 for pole. It means that this area was a high species richness^1^ of vegetation which potentially serve food and nest tree for orangutans. Hopong forest also serve hosts trees in all diameter classes with the most abundant class at 5 cm – 9.9 cm. The distribution of class diameters showed an exponential descending L curve (Fig. 2).

## DISCUSSION

This study confirmed that whilst orangutan are still present in Hopong, the population has declined in the past seven years. This downward trend might be due to human encroachment towards the forest area. Human encroachment leads habitat loss that threats the long-term survival of orangutan. Like most other primate species, orangutan populations are declining over time due to habitat loss and hunting (Estrada *et al.* 2017).

There was significant changing of Hopong forest from 2008 to 2015. Some of the forested areas in 2008 were cleared and converted into settlement, road construction, and agriculture. Along with this, the declining of orangutan and nest encountered was occurred. It shows that orangutan leave areas with high levels of human activity and migrate to the remaining undisturbed forest in Hopong^2^. Reduction of forest cover will reduce fruit availability for orangutans, which can impact the behaviour (Carne *et al.* 2015), in this case, orangutans migrate away from the disturbance area.

Protect the orangutan habitat is mean protect the orangutan population. Re-assesment of government land use policy is needed to protect both habitat and population of orangutan in Hopong. Unprotected forest is vulnerable and their disappearance may further negatively affect orangutan populations here in the future. Unfortunately, most of Hopong forest is gazetted as unprotected forest which vulnerable for habitat loss due to land conversion. Land conversion tends to be more common in unprotected forest which triggers habitat loss and directly impacts orangutan (Gaveau *et al.* 2012). Unsustainable land clearing was found around Hopong forest. The forested area along the edge of the road to main forest was cleared. The villagers cleared the land to establish an ownership claim to it which is indicated from the land that did not managed yet (Sugesty Muhammad Arif, personal communication). The land clearing is estimated to have occured about 1–1.5 years ago.

Hopong forest is a corridor which connects Dolok Sipirok nature reserve and EBTFB. The gazettment as unprotected forest will facilitate land conversion which could cause fragmentation and intrusion into the orangutan home ranges. If it continues like this, the population will disappear by 2030 (Wich *et al*. 2016).

Human-orangutan conflict in Hopong usually occured during the *petai* and durian season (Iman Siagian, personal communication). Primates which live neighbouring with agriculture land raid crops when the food availability in their natural habitat is low (Strum 1994). Human-orangutan conflicts will increase as people expand their agricultural activity and encroach orangutan habitat (Campbell-Smith *et al.* 2010). Crop-raiding is one of the human-orangutan conflict that frequently increased caused by land conversion (Campbell-Smith *et al*. 2011). The villagers consider orangutan a pest species within agricultural areas. Conflict occured because some agricultural crops are overlapped with the orangutan feeding trees, such as durian, *petai*, palm sugar and damar.

The deep forest of Hopong is relatively good forest with natural tree cover. Although some land was cleared at the forest edge, there has been no clearence within the forest. However, we are still concern that land clearing will occur within the forest interior. Hopong forest serve various species vegetation which very important for the orangutan sustainability. The vegetation provide a food source, nesting tree and media for arboreal locomotion for orangutan. Logging will reduce the number of fruits tree which impacted to reduction of fruit availability (Johns & Johns 1995) which at the end will reduced the orangutan density (Rao & van Schaik 1997).

The density of fruit tree and ficus tree are quite low during the study. Five species of fruiting trees were identified in study area, but none of them were fleshy-pulp fruits. Fruit density is seasonal, and varies between species. Most orangutan feed on flushy-pulp fruits, the variation of fruit density influences orangutan density in the short term (Rijksen 1978). This could be one reason why orangutan were not encountered in Hopong. Few ficus trees are present in Hopong. A cluster of big strangler ficus was found away from the transects, but they were not bearing fruits during the sampling time and no orangutan nests were encountered nearby. The trend of fruit and ficus density in this area during the first survey and this study cannot be compared because there were no data for fruit and ficus density collected during the previous survey. On the other hand, the distribution of class diameters showed an exponential descending L curve which means that Hopong potentially being balanced and sustainable forest

This study alarmed the conservation measures for Tapanuli orangutan. The small population size and isolation geographic might lead the inbreeding depression and threat the population existence of this new great apes species (Nater *et al.* 2017).

## CONCLUSION

Orangutan population in unprotected forest tends to follow the downward trend. This indication was shown in the orangutan population of Hopong that has declined in the last seven years. The declining was primarily due to habitat encroachment and land conversion for settlement, road construction, and local agriculture. Hopong forest still has pristine forest that provides a suitable habitat for orangutans. The gazzetment of this forest is extremely required to reduce the threats facing the orangutan population at Hopong.

## Figures and Tables

**Table 1:** Result of first survey (2008) and updated survey (2015).

	2008	2015
Orangutan indication	Direct and indirect	Indirect
Transect length	4.025 km	6 km
N nests (in transect)	43	37
D nests	399/km^2^	234/km^2^
D orangutan	0.7 ind/km^2^	0.4 ind/km^2^
W	0.0134 km	0.0132 km
D fruit	-	0.89 ind/km^2^
D ficus	-	I: 0.11; II: 0

**Table 2:** Vegetation in Hopong.

Species	Family	Important value
Tree level		
Gymnostoma sumatrana*	Casuarinaceae	8.03
*Gordonia oblongifolia*+	Theaceae	7.74
Payena acuminata*	Sapotaceae	6.41
Syzygium chloranthum*	Myrtaceae	4.74
*Palaquium* cf. hexandrum*	Sapotaceae	4.65

	H′	4.5

Pole level		
*Aglaia* sp.*+	Meliaceae	10.93
*Canarium* cf. denticulatum*	Burseraceae	10.34
Dacryodes costata^* +^	Burseraceae	10.08
Gymnostoma sumatrana*	Casuarinaceae	8.42
*Elaecarpus* cf. *parvifolius*	Elaeocarpaceae	7.73

	H′	4.39

*Notes*:

* feeding tree;

+ nest tree

## Footnotes

1 Based on Shannon index: H′<1: low diversity, 1<H′<3: diversity is moderate, H′>3: high diversity (Odum, 1993)

2 Based on interview with the local people, they know that orangutan is protected under the Indonesia Law. First survey also showed that local in this area did not hunting/poaching the orangutans.
